# Priming by Timing: *Arabidopsis thaliana* Adjusts Its Priming Response to Lepidoptera Eggs to the Time of Larval Hatching

**DOI:** 10.3389/fpls.2020.619589

**Published:** 2020-12-09

**Authors:** Georgios Valsamakis, Norbert Bittner, Nina E. Fatouros, Reinhard Kunze, Monika Hilker, Vivien Lortzing

**Affiliations:** ^1^Applied Zoology/Animal Ecology, Institute of Biology, Freie Universität Berlin, Berlin, Germany; ^2^Applied Genetics, Institute of Biology, Freie Universität Berlin, Berlin, Germany; ^3^Biosystematics Group, Wageningen University, Wageningen, Netherlands

**Keywords:** Brassicaceae, insect eggs, Lepidoptera, plant defense, PR genes, priming, salicylic acid

## Abstract

Plants can respond to eggs laid by herbivorous insects on their leaves by preparing (priming) their defense against the hatching larvae. Egg-mediated priming of defense is known for several plant species, including Brassicaceae. However, it is unknown yet for how long the eggs need to remain on a plant until a primed defense state is reached, which is ecologically manifested by reduced performance of the hatching larvae. To address this question, we used *Arabidopsis thaliana*, which carried eggs of the butterfly *Pieris brassicae* for 1–6 days prior to exposure to larval feeding. Our results show that larvae gained less biomass the longer the eggs had previously been on the plant. The strongest priming effect was obtained when eggs had been on the plant for 5 or 6 days, i.e., for (almost) the entire development time of the *Pieris* embryo inside the egg until larval hatching. Transcript levels of priming-responsive genes, levels of jasmonic acid-isoleucine (JA-Ile), and of the egg-inducible phytoalexin camalexin increased with the egg exposure time. Larval performance studies on mutant plants revealed that camalexin is dispensable for anti-herbivore defense against *P. brassicae* larvae, whereas JA-Ile – in concert with egg-induced salicylic acid (SA) – seems to be important for signaling egg-mediated primed defense. Thus, *A. thaliana* adjusts the kinetics of its egg-primed response to the time point of larval hatching. Hence, the plant is optimally prepared just in time prior to larval hatching.

## Introduction

Infestation of plants by herbivorous insects can start harmlessly by deposition of eggs on the plant. From these yet harmless eggs, herbivorous larvae will hatch, and they may heavily damage the plant. However, plants are able to perceive insect egg deposition and to mobilize defense responses killing the eggs. For example, the production of ovicidal substances, the formation of neoplasms or necrotic tissue may result in egg intoxication, detachment of eggs from the leaf, or egg desiccation. Oviposition-induced plant volatiles (OIPVs) and oviposition-induced chemical changes of the leaf surface can attract and arrest egg-killing parasitoids (e.g., Hilker and Fatouros, 2015, 2016; Geuss et al., 2017).

When direct plant defense responses do not kill all eggs or when egg parasitoids are absent, plants remain vulnerable to herbivory by hatching larvae. Then, feeding damage by the larvae can induce defense responses targeting the larvae (Karban and Baldwin, 1997; Howe and Jander, 2008; Mithöfer and Boland, 2012; Stam et al., 2014). The major drawbacks of defense responses induced by insect feeding damage are that it takes some time to scale the defense to full effectiveness against the herbivorous insect and that it is associated with plant fitness costs (e.g., Heil and Baldwin, 2002; Steppuhn and Baldwin, 2008; Cipollini et al., 2017).

To prime, i.e., to prepare for, an inducible defense against impending herbivory by responding to stimuli indicating impending insect infestation is a plant strategy to overcome these drawbacks (Frost et al., 2008). This strategy enables a plant to accelerate the establishment of an effective defense or to amplify anti-herbivore defense responses (Hilker et al., 2016). Thus, primed plants show improved defense, which harms the herbivore to a greater extent than the defense of non-primed plants. Therefore, primed plants might benefit from having lower fitness costs than non-primed plants (Hilker et al., 2016; Hilker and Fatouros, 2016; Martínez-Medina et al., 2016). In general, exposure of a plant to a wide range of biotic and abiotic natural stimuli and also to synthetic compounds may have a priming effect on plant responses to subsequent stress (Mauch-Mani et al., 2017). Naturally occurring environmental stimuli that may reliably indicate impending herbivory and prime plants for improved defense against attack by herbivorous arthropods are, for example, volatile compounds released by herbivorous insects or by herbivore-infested plants. Exposure of plants to insect sex pheromones (Helms et al., 2014, 2017; Bittner et al., 2019), to herbivory-induced plant volatiles (HIPVs, e.g., Heil and Kost, 2006; Dicke and Baldwin, 2010; Karban et al., 2014), or to insect OIPVs (Pashalidou et al., 2020) has been shown to render a plant’s anti-herbivore defense more effective. Furthermore, herbivory preceding further herbivory (e.g., Rasmann et al., 2012) and insect egg deposition preceding larval feeding damage (Hilker and Fatouros, 2015, 2016) are known to enhance plant defenses against the feeding stages of the herbivores.

By now, several studies showed that plant responses to eggs from specialist and generalist insects can prime anti-herbivore defenses against hatching larvae. Among these egg-primable plants are herbaceous annual species (several brassicaceous species, *Nicotiana attenuata*, *Vicia faba*, e.g., Geiselhardt et al., 2013; Pashalidou et al., 2013, 2015; Bandoly et al., 2016; Bonnet et al., 2017; Rondoni et al., 2018; Lortzing et al., 2019; Paniagua Voirol et al., 2020), a perennial shrub (*Solanum dulcamara*; Geuss et al., 2018), and two tree species (*Pinus sylvestris*, *Ulmus minor*; Beyaert et al., 2012; Austel et al., 2016). When larvae feed on previously egg-laden plants, they gain less biomass, suffer higher mortality, need more time to develop and/or have a weaker immune system than larvae feeding on non-primed plants (Bandoly et al., 2016; Hilker and Fatouros, 2016). Egg-mediated improved resistance against feeding larvae has been shown to be attributed to stronger or earlier expression of defense-related genes (Altmann et al., 2018; Lortzing et al., 2019) and to increased levels of phenylpropanoid derivatives that feeding larvae take up (Bandoly et al., 2015, 2016/*N. attenuata*; Austel et al., 2016/*U. minor*; Geuss et al., 2018/*S. dulcamara*; Lortzing et al., 2019/*A. thaliana*; Lortzing et al., 2020). Signaling of egg-mediated priming of anti-herbivore defense has especially been studied with respect to the phytohormones salicylic acid (SA) and jasmonic acid (JA).

Salicylic acid levels of brassicaceous plants (*Brassica nigra*, *A. thaliana*) are induced by egg deposition of the butterfly *Pieris brassicae*. Feeding-damaged plants previously exposed to *P. brassicae* eggs also show higher SA levels than plants only exposed to larval feeding (Bonnet et al., 2017; Lortzing et al., 2019). Further studies revealed that the egg-mediated priming effect of *A. thaliana*’s defense against hatching *P. brassicae* larvae is dependent on SA (Lortzing et al., 2019). This SA dependence has been proven by testing the effect of prior egg deposition on the performance of larvae feeding on mutant plants impaired in SA synthesis, including a *sid2* mutant (Lortzing et al., 2019). *SID2* encodes the isochorismate synthase involved in SA biosynthesis (Wildermuth et al., 2001). Performance of larvae feeding for 48 h on a *sid2* mutant was not affected by the plant’s response to prior egg deposition (Lortzing et al., 2019). The SA-dependent, egg-mediated priming effect on *A. thaliana* defense against *P. brassicae* larvae is also linked with enhanced expression of SA-responsive, pathogenesis-related (*PR*) genes and of a gene encoding a cation exchanger (*CAX3*) and a plant defensin (*PDF1.4*). These genes show higher transcript levels in feeding-damaged, previously egg-laden plants than in feeding-damaged, egg-free ones (Lortzing et al., 2019). Higher transcript levels of *PR* genes and of *PAD3* were also detected in undamaged, egg-laden *A. thaliana* plants than in egg-free ones (Little et al., 2007; Bruessow et al., 2010; Gouhier-Darimont et al., 2013; Paniagua Voirol et al., 2020). *PAD3* encodes a cytochrome P450 enzyme that catalyzes the last step of camalexin biosynthesis in *A. thaliana* (Zhou et al., 1999; Schuhegger et al., 2006). *PAD3* expression is suggested to be both SA-responsive (Glazebrook, 2005; Glawischnig, 2007) and JA-responsive (Pangesti et al., 2016). Several studies indicate that camalexin does not only play a role in plant immunity against phytopathogens but also in plant resistance against herbivory (Pangesti et al., 2016, and references therein). However, whether egg-laden, feeding-damaged plants contain higher camalexin levels than egg-free, feeding-damaged ones is unknown yet.

In spite of the central role of JA and its bioactive conjugate JA-isoleucine (JA-Ile) in plant resistance against chewing herbivores (Wasternack and Hause, 2013; Wasternack, 2015; Lortzing and Steppuhn, 2016), the role of these phytohormones as well as of others like abscisic acid (ABA) in the egg-mediated priming process is not clear yet (Bonnet et al., 2017; Lortzing et al., 2019, 2020). Disentangling their roles is hampered because (i) JA levels change in a strongly time-dependent manner after injury (Koo et al., 2009, and references therein), (ii) JA levels have only been measured at few time points after larval feeding on egg-primed plants, and (iii) other phytohormones than SA and JA have hardly been measured in the context of egg-mediated anti-herbivore defense-priming (compare Lortzing et al., 2019). Nevertheless, hints on the relevance of JA in egg-mediated priming of plant resistance against feeding larvae have been provided by studies on solanaceous species. Tomato plants (*Solanum lycopersicum*), which received egg depositions of the moth *Helicoverpa zea*, showed enhanced JA levels in response to subsequent wounding and application of oral secretion of conspecific larvae (Kim et al., 2012). This effect was detectable early after the application of oral secretion, i.e., after 30 and 60 min, but not later. However, an adverse effect of prior egg deposition on *H. zea* larvae feeding on the tomato plants was not shown. In *N. attenuata*, the transcription factor MYB8 plays a crucial role in egg-mediated priming of enhanced resistance against *Spodoptera exigua* and *Manduca sexta* larvae (Bandoly et al., 2015, 2016). MYB8 is activated in response to JA-mediated induction by *M. sexta* larval herbivory (Onkokesung et al., 2012). However, JA levels in egg-primed, feeding-induced plants were not higher than in non-primed, feeding-induced plants when measured 1 day after wounding (Bandoly et al., 2015; Drok et al., 2018).

Most studies on egg-mediated priming of plant defense against herbivores quantified resistance traits of plants exposed to insect eggs over the natural time needed by the embryo inside the egg to develop until larval hatching. For example, at moderate temperature (20–21°C), *P. brassicae* larvae hatch from eggs 6 days after oviposition on *A. thaliana* leaves. Neonate larvae feeding for at least 48 h on previously egg-laden plants show worse performance than larvae on egg-free plants (Geiselhardt et al., 2013; Lortzing et al., 2019; Paniagua Voirol et al., 2020). The priming effect of prior egg deposition is not only obvious by impaired larval development but also by less feeding damage upon egg-primed plants (Geiselhardt et al., 2013).

Up to now, little is known about the kinetics of expression of priming-relevant defense genes and the phytohormone levels during the natural egg-priming phase and how this affects the subsequently feeding larvae. For *A. thaliana* it is shown that SA levels and transcript levels of *PR1* and *PR5* increase over a period of 3–4 days after *P. brassicae* egg deposition or treatment with egg extracts (Little et al., 2007; Bruessow et al., 2010; Gouhier-Darimont et al., 2013). However, whether the kinetics of these and other priming-relevant defense traits is optimally adjusted to the time point of larval hatching has not been investigated so far.

To address the above-mentioned gaps in knowledge, we used *A. thaliana* and *P. brassicae* as the study system. We investigated (i) for how long eggs need to remain on a plant until a significant priming effect on plant defense against hatching larvae is reached. We further studied (ii) changes in expression of defense genes and phytohormone levels in dependence of the time past egg deposition and the duration of larval feeding. We measured larval performance as proxy of plant resistance, quantified transcript levels of defense-related genes and of genes involved in phytohormone biosynthesis and signaling, and measured phytohormone concentrations. Furthermore, (iii) we quantified levels of camalexin in egg-laden and feeding-damaged plants. We investigated (iv) the role of camalexin and of JA-Ile in egg-mediated priming of *A. thaliana* defense against larvae by analyzing larval performance on egg-laden mutant plants impaired in the biosynthesis of camalexin and JA-Ile, respectively. We hypothesized that camalexin accumulates in response to the eggs and thus negatively affects performance of neonate larvae.

We show that *A. thaliana* needs to perceive *P. brassicae* eggs for almost the entire egg incubation time (5–6 days) to mount a response that results in improved (primed) defense against hatching larvae. During the egg priming period, plants responded with distinct expression patterns of defense-related genes and induction of phytohormones that may contribute to the reinforced anti-herbivore defense response. Our results further suggest that not only SA but also other phytohormones, including JA-Ile, might play a role in egg-mediated priming of defense against the larvae, whereas the egg-inducible camalexin does not affect the performance of the larvae.

## Materials and Methods

### Plant Material

*Arabidopsis thaliana* Columbia-0 (Col-0) wild type (WT) and mutant plants were grown as described by Firtzlaff et al. (2016) under short-day conditions (8 h/16 h light/dark cycle, 120 μmol m^−2^ s^−1^ light intensity, 20°C, and 50% relative humidity). The mutant *sid2* (SALK_088254) was established in our lab, and the mutant *jar1-1* was kindly provided by Anne Cortleven (Freie Universität Berlin). Mutant *pad3-1* was obtained from the European Arabidopsis Stock Centre (http://arabidopsis.info), originally established by Glazebrook et al. (1997). The plants were treated in the vegetative stage when they were 6–7 weeks old.

### Insect Rearing

Adults of the Large Cabbage White Butterfly, *P. brassicae* (Lepidoptera: Pieridae), were reared in flight cages (45 cm × 45 cm × 60 cm) in a climate chamber under long-day conditions (18 h/6 h light/dark cycle, 220 μmol m^−2^ s^−1^ light intensity, 23°C, and 70% relative humidity). Butterflies were fed with a fresh 15% aqueous honey solution every 2–3 days. Mated females were allowed to lay eggs on Brussels sprouts plants (*Brassica oleracea* var. *gemmifera*). Plants laden with eggs were kept in a cage in another climate chamber (18 h/6 h light/dark cycle, 160 μmol m^−2^ s^−1^ light intensity, 20°C, and 70% relative humidity) until the larvae hatched. Larvae remained in the same climate chamber and fed on Brussels sprouts throughout their entire larval development until pupation.

### Experimental Setup and Plant Tissue Sampling

#### Experimental Setup I

Experimental setup I was designed to determine (a) for how long eggs need to remain on a plant until a priming effect on larval performance is detectable and (b) transcription levels as well as phytohormone and camalexin concentrations in leaves depending on the time of plant exposure to eggs.

**Figure 1:**
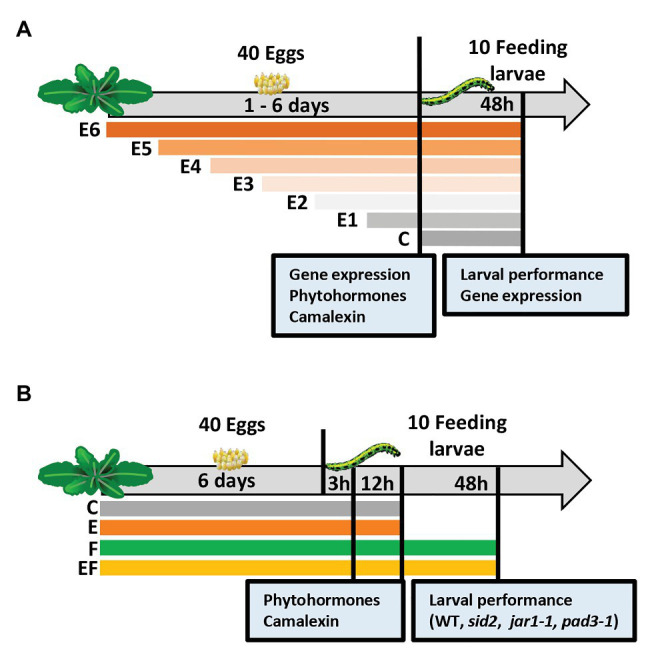
Experimental setups studying timing of priming of *Arabidopsis thaliana* anti-herbivore defense by prior *Pieris brassicae* egg deposition and the effects of induced levels of phytohormones and camalexin on larval performance. **(A)** Experimental setup I focusing on the analysis of plant responses to insect eggs and larval feeding in dependence of the time period, for which plants are exposed to eggs: we determined the effect of *P. brassicae* egg deposition on *A. thaliana* plant gene expression, phytohormones, and camalexin levels after different egg exposure times: 1 day (E1), 2 days (E2), 3 days (E3), 4 days (E4), 5 days (E5), or 6 days (E6), and after 48 h of larval feedings. In addition, larval biomass and plant gene expression were determined 48 h after larval feeding. C = control plant without eggs. **(B)** Experimental setup II focusing on changes of plant responses early after the onset of larval feeding (3 h, 12 h) upon plants, which experienced egg exposure times for 6 days: we determined the effect of prior *P. brassicae* egg deposition on phytohormone and camalexin levels and the effect of prior egg deposition on performance of larvae feeding upon *A. thaliana* mutant lines. For phytohormone and camalexin quantification, the plants were exposed to eggs for 6 days (E), larval feeding (F), eggs and subsequent larval feeding (EF), or plants were left untreated (C). Leaf material was harvested after a 3 h or 12 h feeding period. Larval performance was evaluated after 48 h feeding on wild type (WT) plants or on SID2-, JAR1-, or PAD3-deficient/impaired mutant lines.

Each *A. thaliana* plant was exposed to one *P. brassicae* egg cluster consisting of 40 ± 5 eggs. The butterfly was allowed to lay this egg cluster on rosette leaves 14–17. The plants were exposed to eggs for 1 day (E1), 2 days (E2), 3 days (E3), 4 days (E4), 5 days (E5), or 6 days (E6), or left untreated as controls (C; Figure 1A). For each plant that received eggs, different females were used, thus providing independent biological replicates. At the end of the egg exposure time, the egg cluster was gently peeled off the leaf with a pair of tweezers. Thereafter, the leaf was harvested for gene expression, phytohormone or camalexin analysis, or neonate *P. brassicae* larvae were placed on the previously treated leaf.

To obtain feeding-damaged plants, 10 neonate *P. brassicae* were placed onto the leaf that previously had carried eggs (EF plants) or to egg-free leaves of previously untreated plants (F plants). The larvae had hatched from eggs laid on non-experimental *A. thaliana* plants kept in a climate chamber (8 h/16 h light/dark cycle, 120 μmol m^−2^ s^−1^ light intensity, 20°C, and 50% relative humidity). One day before larval hatching, the eggs were carefully removed from the non-experimental plants. The eggs were subsequently kept in Petri dishes in the same climate chamber. After larval hatching on the following day, the neonates were placed into clip cages (2 cm in diameter, 1.7 cm high). The clip cages were mounted to the leaf with the former egg cluster (EF plants) or to leaves of egg-free plants with similar leaf position within the plant rosette (F plants). For control, we mounted empty clip cages on leaves of egg-free plants, i.e., C plants, and on E plants, which had received eggs but were not exposed to larvae.

The experimental setup I was used for three experiments. In one of them, we analyzed larval performance, in another independent one, we measured plant gene expression levels, and in a third independent one, phytohormone and camalexin levels were measured.

To determine the effect of different egg exposure times on the performance of *P. brassicae* larvae, we let larvae feed for 48 h on the plants, and then larval biomass was measured (see below). For control, we also determined the biomass of larvae feeding for 48 h on egg-free plants (Figure 1A).

For gene expression analysis, phytohormone and camalexin quantification, leaf material was harvested from the different plant treatments at the end of the egg exposure period. Since the E6 treatment was done first, followed by the E5 treatment 1 day later, etc., leaf material from all plants could be harvested on the same day (Figure 1A). For control, we also analyzed untreated control plants.

We quantified levels of the phytohormones SA, JA, JA-Ile, and ABA, as well as of the phytoalexin camalexin in plants exposed to eggs for 1–6 days and in untreated control plants (Figure 1A).

In addition, leaf material from feeding-damaged samples without prior egg deposition or with prior egg deposition (for 1–6 days) was harvested for gene expression analysis. These plants were exposed to feeding by neonate larvae for 48 h.

#### Experimental Setup II

A second experimental setup was designed to study the kinetics of changes in phytohormone levels and camalexin early after the onset of larval feeding. We studied the phytohormones SA, JA, JA-Ile, and ABA. We used a full factorial setup with the following treatments: (C) untreated controls, (E) 6 days egg deposition by *P. brassicae*, (F) feeding damage by *P. brassicae* larvae, and (EF) 6 days egg deposition followed by larval feeding.

Treatment of plants with eggs was conducted as described for experimental setup I, but for setup II, the eggs always remained on the plant for 6 days. Treatment of plants with larvae was conducted also as described for the experimental setup I, but here larvae were allowed to feed either for 3, 12, or 48 h (Figure 1B).

To quantify phytohormones and camalexin, leaf material was harvested from the different plant treatments after 3 and 12 h of feeding, and after respective time periods from untreated plants or undamaged, egg-laden plants (Figure 1B).

To elucidate the relevance of SA, JA, and camalexin in egg-mediated priming of *A. thaliana* resistance against *P. brassicae* larvae, we compared larval biomass on egg-free and egg-laden WT plants with the larval biomass on egg-free and egg-laden mutant plants impaired in SA biosynthesis (*sid2*), in conjugating JA to JA-Ile (*jar1-1*), or in camalexin biosynthesis (*pad3*-1). The larval biomass was measured after a 48 h feeding period (Figure 1B) in three independent experiments; one compared larval biomass on WT plants, *sid2* plants, and *jar1-1* plants, another one was done for verifying the results obtained with *jar1-1* plants and WT plants, and a third experiment was conducted to compare larval biomass on WT plants with larval biomass on *pad3-1* plants.

#### Larval Performance

The average biomass per larva was calculated for each plant replicate independently. The total biomass of all feeding larvae on each plant replicate was determined on a Sartorius MSA125P-100-DI Cubis Semi-Micro Balance (Sartorius Lab Instruments GmbH and Co, Göttingen, Germany) and subsequently divided by the number of larvae feeding on the plant so that the average biomass per larva per plant replicate was calculated. Thereafter, the mean larval biomass was calculated for each plant treatment.

#### RNA Extraction and Quantitative Real-Time PCR

Total RNA was extracted from leaf material as described by Oñate-Sánchez and Vicente-Carbajosa (2008). Residual genomic DNA was removed with TURBO DNA free™ kit (ThermoFisher Scientific, Waltham, United States). For first-strand cDNA synthesis, 2 μg total RNA in 10 μl reactions were reverse transcribed with the RevertAid First Strand cDNA Synthesis Kit (ThermoFisher Scientific) following the manufacturer’s protocol. Quantitative real-time PCR (qPCR) was conducted on a Stratagene MX3005p Real-Time PCR System (Stratagene Systems, Washington, United States) in 10 μl reactions with 1 μl cDNA, 0.5 μl of each gene-specific primer (2.5 μM), and 5 μl Power SYBR® Green PCR master mix (Applied Biosystems) with the following thermal profile: 1 × 10 min 95°C − 40 × (90 s 95°C − 60 s 60°C) followed by melt curve analysis at 95°C for 60 s to 60°C for 30 s to 95°C for 30 s. Samples were checked for genomic DNA residues with primers specific for genomic DNA.

We determined the expression levels of a set of genes known to be (i) inducible by insect egg deposition, involved in (ii) egg-mediated priming, (iii) phytohormone signaling and biosynthesis, and (iv) camalexin biosynthesis. As reference genes, we used *AtACT2* (At3g18780), *UBQ10* (At4g05320), and *GAPDH* (At1g13440; Kozera and Rapacz, 2013). Supplementary Table S1 provides a list of the analyzed genes and information on the primer sequences used for the transcript analysis. Relative expression levels were calculated according to Livak and Schmittgen (2001).

#### Phytohormone and Camalexin Analysis

Camalexin and the phytohormones SA, JA, JA-Ile, and ABA were extracted based on the protocol from Wang et al. (2007). In detail, leaf tissue was harvested in 2 ml tubes with homogenization matrix (Zirconox, 2.8–3.3 mm, Mühlmeier Mahltechnik, Bärnau, Germany) and frozen in liquid nitrogen. We added 1 ml ethyl acetate with 2 μl internal standard mix to each sample. The standard mix contained deuterated phytohormones, i.e., 10 ng/μl D4-SA, 10 ng/μl D6-abscisic acid (OlChemIm Ltd., Olomouc, Czech Republic), 30.2 ng/μl D6-JA, and 10 ng/μl D6-JA-Ile (HPC Standards GmbH, Cunnersdorf, Germany). The sample with these additions was homogenized for 3 × 20 s at 6 m s^−1^ in a grinder (Bertin technologies Precellys® Evolution, Montigny-le-Bretonneux, France). Homogenates were centrifuged at 4°C and 13,000 *g* for 10 min in an Eppendorf® centrifuge 5427R with rotor FA-45-48-11 (Eppendorf AG, Hamburg, Germany). Supernatants were transferred to new tubes. The extraction procedure was repeated with ethyl acetate without internal standard mix. Supernatants were combined and concentrated in an Eppendorf Concentrator 5301. Re-elution of phytohormones in 300 μl 70% methanol with 0.1% formic acid (v/v) was performed under vortexing for 10 min at room temperature (RT; Scientific Industries, model: Vortex-Genie 2 T, Bohemia New York, United States). Samples were centrifuged for at least 20 min at 13,000 g RT. The supernatant was transferred to HPLC vials (200 μl) and stored at −20°C until measurement.

Phytohormones and camalexin were separated, detected, and quantified by using UPLC-MS/MS (Q-ToF-ESI; Synapt G2-S HDMS; Waters®, Milford, Massachusetts, United States). Seven microliters extract were injected into the UPLC system (AQUITY™, Waters, Milford, Massachusetts, United States). Phytohormones and camalexin were separated on a C_18_ column (Acquity UPLC Waters, BEH-C18, Ø 2.1 mm × 50 mm, particle size 1.7 μm) using water and methanol [each with 0.1% formic acid (v/v)] as eluents A and B, respectively, in a gradient mode with a constant flow of 250 μl min^−1^ at 30°C: eluent B: 0 min 30%; 1 min 30%; 4.5 min 90%; 8 min 90%; 9 min 30%; and 3 min equilibration time between the runs. Separated compounds were negatively ionized by electrospraying (ESI) using the following conditions: capillary voltage 2.5 kV, nebulizer 6 bar, desolvation gas flow rate 500 l/h, 80°C source temperature and 150°C desolvation temperature, and N_2_ as desolvation gas. The compounds were detected by tandem mass spectrometry, and the full compound mass spectrum was scanned between 50 and 600 *m/z*. The compound annotation was based on the characteristic parent [M–H]-ion and a diagnostic daughter ion, and for phytohormones additionally on co-elution with their deuterated derivatives. The characteristic ions for the analyzed compounds were for camalexin (*m/z* 199 and 141), SA (*m/z* 137 and 93), ABA (*m/z* 263 and 153), JA (*m/z* 209 and 59), JA-Ile (*m/z* 322 and 130) and for their deuterated derivatives D4-SA (*m/z* 141 and 97), D6-ABA (*m/z* 269 and 159), D6-JA (*m/z* 215 and 59), D6-JA-Ile (*m/z* 328 and 136). For the quantification of the peak areas, we used the MassLynxTM Software (version 4.1; Waters). The phytohormones were quantified *via* the peak areas of the fragment ions relative to the fragment ions of the internal standard. Camalexin was quantified according to the peak area of the fragment ions of the plant-derived camalexin relative to the fragment ions of the external standard using the following dilution series: 0, 0.1, 0.5, 1, 5, 7.5, 10, and 50 μM [M (camalexin) = 200.26 g mol^−1^]. The concentrations of compounds per sample were normalized to the fresh weight.

#### Statistical Analysis

Datasets were statistically evaluated and visualized with the software “R (version 4.0.0)” (R Development Core Team, 2016) and R Studio (version 1.2.5042, R Studio Team, 2020) with the packages “car” (Fox and Weisberg, 2019), “lme4” (Bates et al., 2015), “lmtest” (Zeileis and Hothorn, 2002), “multcomp” (Hothorn et al., 2008), “nlme” (Pinheiro et al., 2020), and “psych” (Revelle, 2020). Normal distribution of data and their variance homogeneity were evaluated with the Shapiro-Wilk and Levene’s test, respectively, and with boxplots. If data were not normally distributed, data were log-transformed to fulfill the criteria for parametric test procedures. The following statistical tests were used: ANOVA with Tukey test for *post hoc* comparison, pairwise *t*-test with Benjamini Hochberg correction, and Student’s *t*-test and linear mixed model with general linear hypothesis test with Tukey contrasts using plant treatment as a fixed factor and experimental block as a random factor.

## Results

### The Longer Insect Eggs Remain on Leaves, the Less Biomass the Feeding Larvae Gain

To assess for how long *P. brassicae* eggs need to stick to the plants until a significant priming effect on plant defense against hatching larvae is reached, we exposed *A. thaliana* for 1–6 days to eggs.

**Figure 2:**
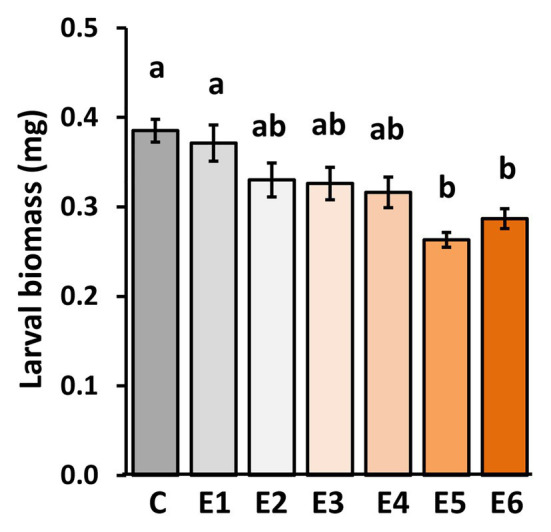
Impact of *P. brassicae* egg deposition period on performance of larvae feeding on the previously egg-laden *A. thaliana* plants. Larval biomass of *P. brassicae* in mg (mean ± SE) after feeding for 48 h on egg-free *A. thaliana* plants control plants (C) or on plants exposed to eggs for 1 day (E1), 2 days (E2), 3 days (E3), 4 days (E4), 5 days (E5), or 6 days (E6). Different lowercase letters above the bars indicate significant differences at the level of *p* < 0.05 (ANOVA, *post hoc* Tukey). Biological replicates (plants) per treatment: *N* = 9–10.

When larvae fed for 48 h on plants that had previously been exposed for 5 or 6 days to *P. brassicae* eggs (E5 and E6), they gained significantly less biomass than the larvae that fed on egg-free plants (C) or on plants exposed for 1 day to eggs (E1; Figure 2). When larvae fed on plants that had previously been exposed to eggs for 2, 3, or 4 days (E2, E3, or E4), they did not gain significantly less biomass than larvae on egg-free plants (Figure 2).

Thus, *P. brassicae* eggs need to remain for at least 5 days on a plant to induce a significantly primed resistance response against larvae.

### The Longer Insect Eggs Remain on the Leaves, the Stronger the Expression of Salicylic Acid- and Priming-Responsive Genes

**Figure 3:**
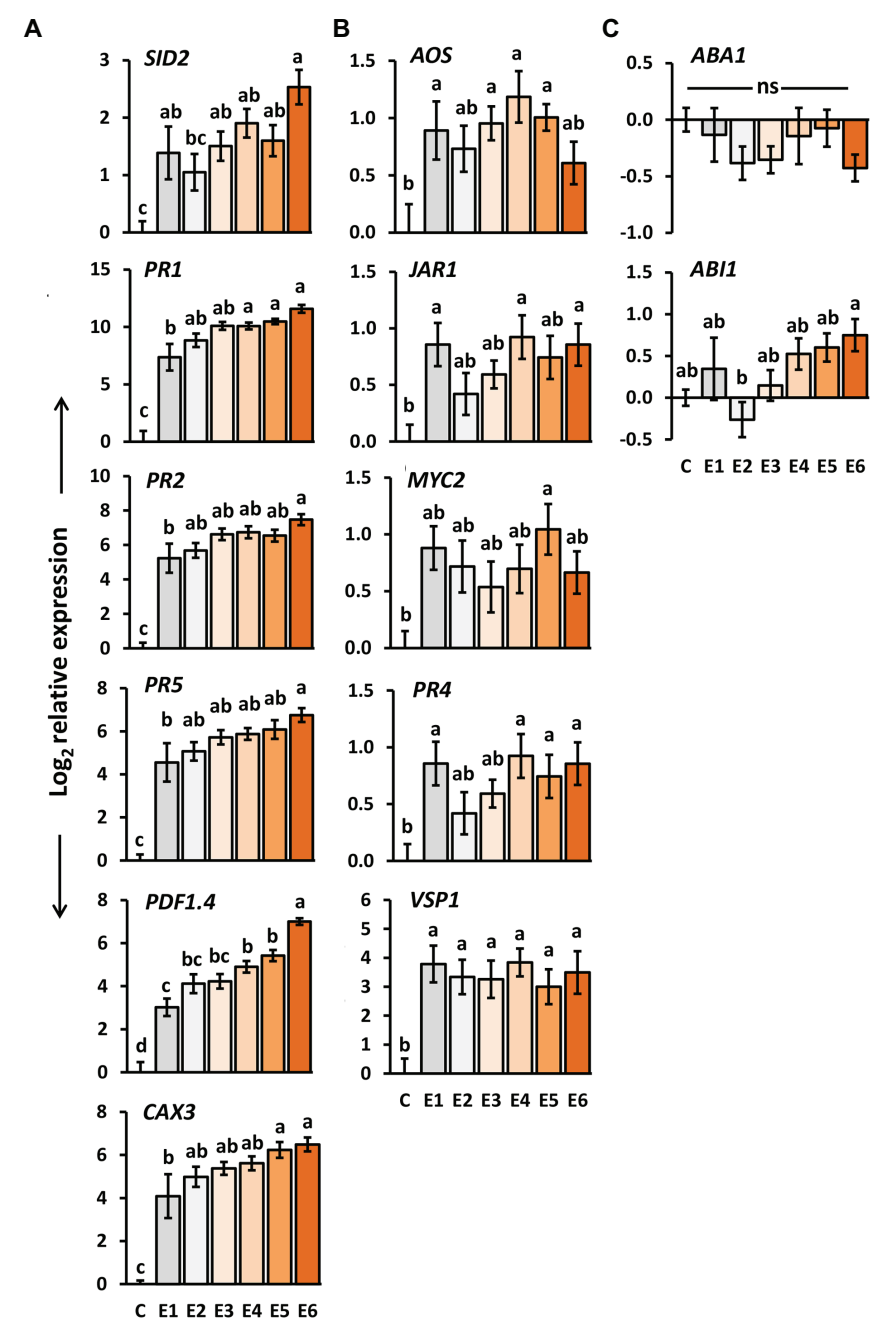
Impact of *P. brassicae* egg deposition period on transcript levels in *A. thaliana* leaves. Relative gene expression (log_2_, mean ± SE) of genes known to be involved in **(A)** egg-mediated responses of *A. thaliana*, **(B)** jasmonic acid (JA) biosynthesis and responsive genes, **(C)** abscisic acid (ABA) biosynthesis and responsive genes in untreated plants (C) and plants exposed for 1 day (E1), 2 days (E2), 3 days (E3), 4 days (E4), 5 days (E5), or 6 days (E6) to *P. brassicae* eggs. Different letters indicate significant differences between treatments (*p* < 0.05, linear mixed model and *post hoc* general linear hypothesis test with Tukey contrasts). Biological replicates (plants) per treatment: *N* = 9–10.

We determined the expression levels of genes in *A. thaliana* plants exposed to *P. brassicae* egg deposition for 1–6 days. *SID2*, *PR1*, *PR2*, *PR5*, *CAX3*, and *PDF1.4* are genes known to be induced by *P. brassicae* eggs and to play a role in egg-mediated priming of *A. thaliana* anti-herbivore defense (Little et al., 2007; Bruessow et al., 2010; Gouhier-Darimont et al., 2013; Lortzing et al., 2019; Paniagua Voirol et al., 2020). *SID2* is involved in SA biosynthesis (Wildermuth et al., 2001), whereas *PR1*, *PR2*, and *PR5* act downstream of the SA signaling pathway (Thomma et al., 1998). *CAX3* encodes for a cation exchanger (Manohar et al., 2011), and *PDF1.4* is suggested to encode a PR protein belonging to a plant defensin family (TAIR-https://www.arabidopsis.org/). The expression levels of all these genes were significantly induced in *A. thaliana* by *P. brassicae* eggs already 1 day after egg deposition on the plant (Figure 3A). The transcript levels gradually increased the longer the eggs remained on the leaves, i.e., the highest expression was reached 6 days after egg deposition.

We also determined the expression of the same genes after a 48 h larval feeding period upon plants that had previously been exposed to egg deposition for 1–6 days. Except for *SID2*, also in feeding-damaged plants, the expression levels of the above-mentioned genes increased with the duration of prior egg exposure. Expression of *SID2* decreased by trend; however, the expression levels in feeding-damaged plants with prior egg deposition did not differ from those in feeding-damaged, egg-free plants after 48 h of feeding (Supplementary Figure S1A).

### Insect Eggs Induce Expression of Jasmonic Acid-Responsive Genes

We investigated how the expression of several genes involved in JA- or ABA-mediated signaling changes after egg deposition by *P. brassicae*. We selected *AOS*, which encodes for allene oxide synthase and is involved in JA biosynthesis in the chloroplasts (Hickman et al., 2017), *JAR1*, encoding an enzyme which conjugates JA with isoleucine (Staswick and Tiryaki, 2004), and *MYC2*, a transcription factor that plays a central role in JA-dependent signaling (Pozo et al., 2008). *PR4* and *VSP1* act downstream of JA signaling in interaction with other phytohormones (Thomma et al., 1998; Berger et al., 2002). As ABA biosynthesis and ABA-responsive genes, we selected *ABA1* and *ABI1*, respectively (Xiong and Zhu, 2003).

The expression of *AOS*, *JAR1*, *PR4*, and *VSP1* was significantly induced in *A. thaliana* 1 day after *P. brassicae* egg deposition (Figure 3B). The expression of *MYC2* was significantly induced only 4 days after egg deposition, but neither after a shorter nor a longer egg exposure period. Egg deposition induced *VSP1* evenly strong over the entire egg incubation period.

No such steady induction was observed for the other genes involved in the JA-mediated signaling network (Figure 3B). Their egg-induced expression was rather low and hardly exceeded a log_2_-fold change in expression greater than one relative to untreated controls. Interestingly, except for *PR4*, expression of these genes was by trend reduced after 48 h larval feeding on egg-laden plants. *PR4* showed a tendency toward upregulation with increasing egg deposition time prior to feeding damage (Supplementary Figure S1B).

The expression levels of the ABA biosynthesis gene *ABA1* and of the ABA-responsive *ABI1* were not significantly affected by *P. brassicae* eggs when compared to egg-free control plants (Figure 3C). Neither did they differ between feeding-damaged plants with and without prior egg deposition (Supplementary Figure S1C).

Altogether, we found the inducibility of JA-responsive genes, but not of ABA-responsive genes by *P. brassicae* egg deposition. The temporal induction pattern of the JA-responsive genes independent of the time past egg deposition differed from the pattern detected for the genes involved in SA biosynthesis and SA-mediated signaling.

### Plant Response to Insect Eggs Results in Increased Levels of SA, JA, and JA-Ile

**Figure 4:**
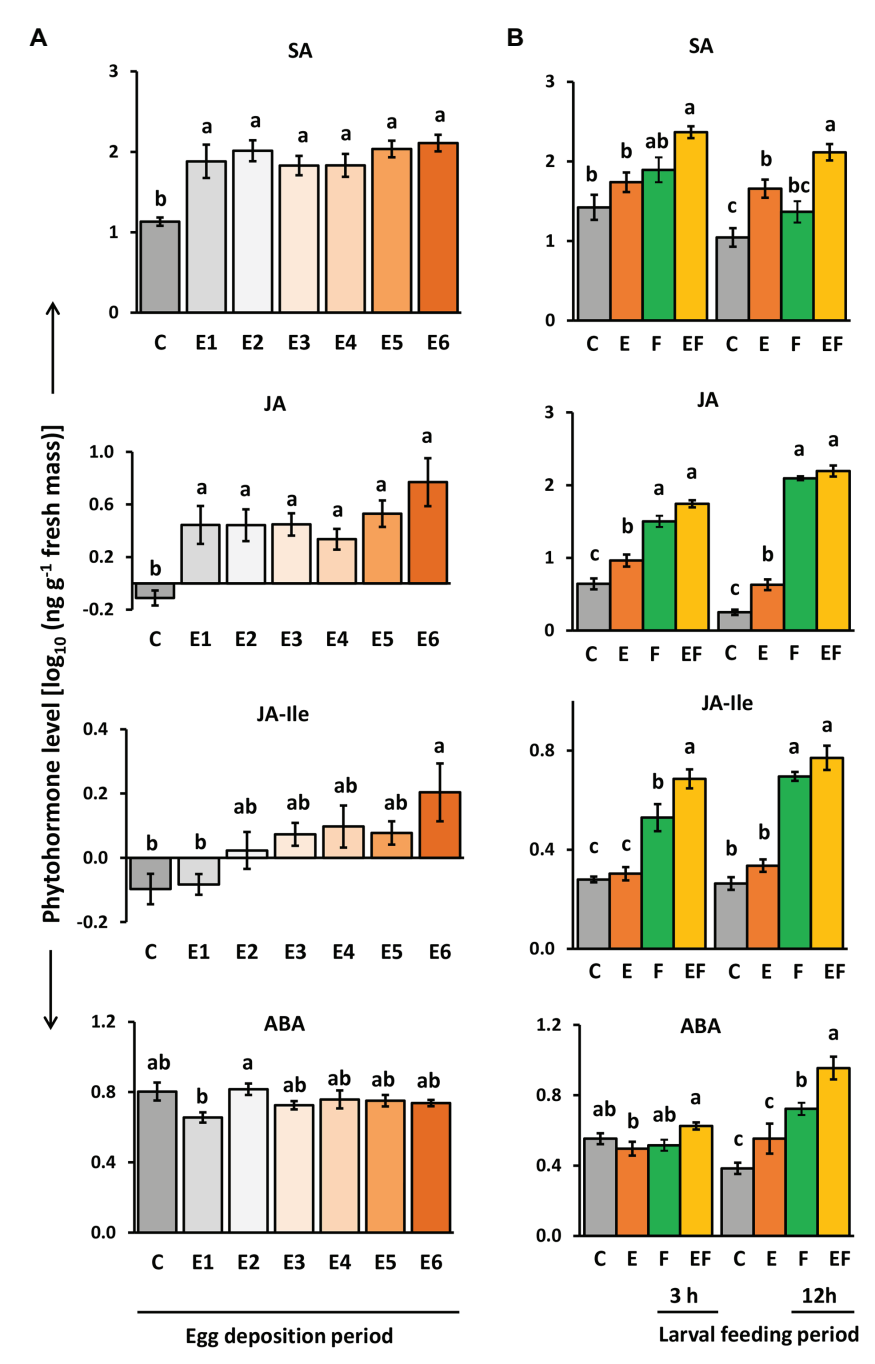
Impact of *P. brassicae* egg deposition on phytohormone levels of non-damaged and larval feeding-damaged *Arabidopsis thaliana* leaves. **(A)** Impact of *P. brassicae* egg deposition period on phytohormone levels and **(B)** impact of 6 days lasting egg deposition on phytohormone levels after a 3 h or 12 h larval feeding period. **(A)** Phytohormone levels in egg-free control plants (C) or in plants exposed to eggs for 1 day (E1), 2 days (E2), 3 days (E3), 4 days (E4), 5 days (E5), or 6 days (E6). **(B)** Phytohormone levels in untreated control plants (C), for 6 days egg-laden plants and subsequent egg removal (E), 3 h or 12 h larval feeding-damaged plants without prior egg deposition (F) and with prior egg deposition for E6 (EF). Concentrations are log_10_-transformed in ng g^−1^ fresh weight (mean ± SE). From top to down: levels of salicylic acid (SA), JA, JA-isoleucine (JA-Ile), and ABA. Different letters indicate significant differences between treatments (*p* < 0.05, linear mixed model and *post hoc* general linear hypothesis test with Tukey contrasts). Biological replicates (plants) per treatment: *N* = 7–10.

To assess if and how levels of SA, ABA, JA, and JA-Ile are affected in *A. thaliana* by *P. brassicae* egg deposition throughout the natural egg deposition period, we quantified the phytohormones with LC/MS (Figure 4A). We further determined how their levels are affected after 3 and 12 h of feeding by *P. brassicae* larvae on egg-free plants or on plants that had previously been exposed to the eggs for 6 days (Figure 4B).

Salicylic acid levels were induced by *P. brassicae* egg deposition and remained constantly high over the egg deposition period of 6 days (Figure 4A). Significantly egg-induced SA levels were also detectable 12 h after removal of eggs that had been on the plant for 6 days and by trend 3 h after egg removal (Figure 4B, E vs. C). When *P. brassicae* larvae fed for 3 or 12 h on a plant that had been exposed to eggs for 6 days, the SA levels were higher than in feeding-damaged, egg-free controls (Figure 4B, EF vs. F). When larvae fed on egg-free plants for 3 or 12 h, SA levels increased by trend, but not significantly (Figure 4B, F vs. C).

Jasmonic acid levels slightly but significantly increased in response to *P. brassicae* eggs. The highest level was reached 6 days after egg deposition (Figure 4A). Egg-induced JA levels were still detectable 3 and 12 h after egg removal (Figure 4B, E vs. C). As expected, JA levels were induced by larval feeding already after a feeding period of 3 h, but the levels between feeding-damaged plants with and without prior egg deposition were neither significantly different after 3 h nor after 12 h of feeding (Figure 4B, EF vs. F).

Jasmonic acid-isoleucine levels followed a similar pattern as the levels of JA, but in contrast to JA, the JA-Ile levels were not induced already 1 day after egg deposition. Instead, plants needed to be exposed for at least 6 days to *P. brassicae* eggs to reach significantly induced JA-Ile levels (Figure 4A). JA-Ile levels were no longer induced after removing the eggs, which had been on the plant for 6 days (Figure 4B, E vs. C). As expected, JA-Ile levels were induced by larval feeding in both egg-free and previously egg-laden plants (Figure 4B, F vs. C and EF vs. C). Interestingly, after a 3 h feeding period, JA-Ile levels were significantly higher in plants that had previously received eggs for 6 days than in egg-free, feeding-damaged plants (Figure 4B, EF vs. F). This difference vanished after a 12 h lasting larval feeding period.

Levels of ABA were not affected by *P. brassicae* egg deposition; no significant change was detected at any egg exposure period in comparison to egg-free plants (Figure 4A). However, after 12 h of larval feeding, ABA levels were induced both in egg-free and previously egg-laden plants (Figure 4B, F vs. C and EF vs. C). These feeding-induced ABA levels were higher in previously egg-laden than egg-free plants (Figure 4B, EF vs. F).

Thus, plants responded to insect eggs with simultaneous induction of SA and JA already 1 day after egg deposition, whereas JA-Ile levels peaked in egg-laden plants just before larvae hatched. Early after the onset of feeding damage, the plant’s response to prior egg deposition resulted in increased feeding-induced JA-Ile and ABA levels.

### The Egg-Mediated Priming Effect on Larvae Is Absent in JAR1-Impaired *Arabidopsis thaliana*

The increase of JA-Ile levels after a 6-day lasting egg exposure period and the egg-mediated enhancement of the feeding-induced JA-Ile levels after a 3 h larval feeding period prompted us to investigate whether JA-Ile is required for egg-mediated priming of plant defense. Therefore, we measured larval biomass after a 48 h feeding period on egg-free *jar1-1* mutants and on *jar1-1* mutants laden with eggs for 6 days. As positive controls, we measured the larval biomass on egg-laden and egg-free WT plants. Furthermore, larval biomass was determined on egg-laden and egg-free *sid2* mutants, which served as negative controls because a previous study revealed that the egg-mediated priming effect on WT plant defense against larvae is abolished when plants are SID2-deficient (Lortzing et al., 2019).

**Figure 5:**
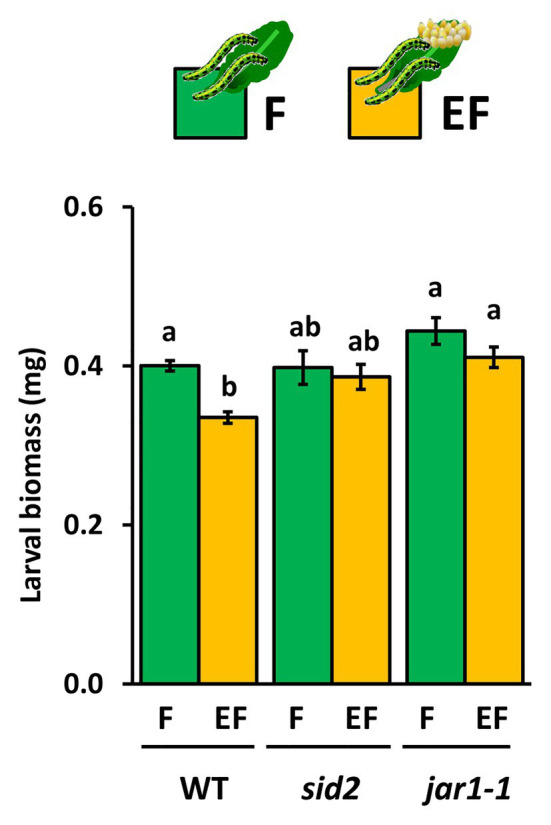
Impact of *Arabidopsis thaliana* JAR1- and SID2-impairment on performance of *P. brassicae* larvae feeding on previously egg-laden plants. Biomass in mg (mean ± SE) of *P. brassicae* larvae after feeding for 48 h on egg-free (F, green) or egg-laden (EF, yellow) WT *A. thaliana* plants, *sid2*- or *jar1–1* mutant lines. Different lowercase letters above the bars indicate significant differences between the treatments at the level of *p* < 0.05 (ANOVA, *post hoc* Tukey). Biological replicates (plants) per treatment: *N* = 10.

Our results show that larvae, which fed for 48 h on previously egg-laden WT plants, gained less biomass than larvae on egg-free plants. The egg-mediated effect on larval biomass was absent in *sid2* and *jar1-1* mutants (Figure 5). The experiment was repeated with WT and *jar1-1* plants showing similar results (Supplementary Figure S2). These results indicate that next to SA also JA-Ile might play a role in egg-mediated priming of inducible plant defense against larvae.

### Camalexin Levels Are Induced by Egg Deposition but They Do Not Affect Priming of Defense Against the Larvae

**Figure 6:**
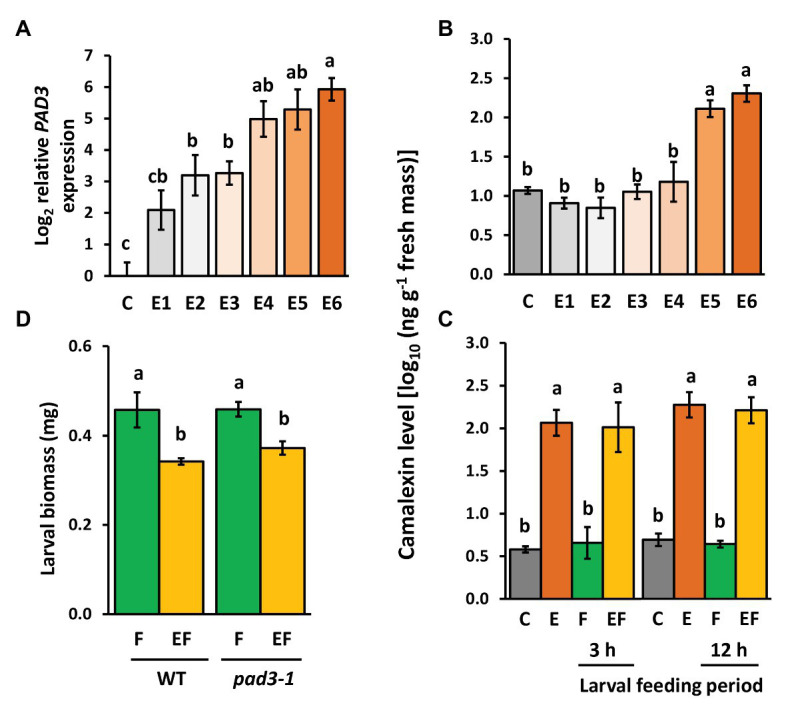
Effect of *P. brassicae* egg deposition and larval feeding on regulation of camalexin levels in *Arabidopsis thaliana*. **(A)** Relative expression of *PAD3* (log_2_, mean ± SE) and **(B)** camalexin levels in egg-free control plants (C) or in plants exposed to eggs for 1 day (E1), 2 days (E2), 3 days (E3), 4 days (E4), 5 days (E5), or 6 days (E6). **(C)** Camalexin levels in untreated control plants (C), for 6 days egg-laden plants (E) and subsequent egg removal, 3 h or 12 h larval feeding-damaged plants without (F) and with prior egg deposition for 6 days (EF). Concentrations of camalexin are log_10_-transformed in ng g^−1^ fresh weight (means ± SEM). **(D)** Biomass in mg (mean ± SE) of *P. brassicae* larvae that fed on previously egg-laden plants (EF) or egg-free (F) WT and *pad3* mutant plants, respectively. Different lowercase letters above the bars indicate significant differences (*p* < 0.05, linear mixed model and *post hoc* general linear hypothesis test with Tukey contrasts). Biological replicates (plants) per treatment: *N* = 8–10.

Expression levels of *PAD3* encoding an enzyme relevant for camalexin biosynthesis increased with increasing time after egg deposition (Figure 6A). Camalexin levels were induced by *P. brassicae* eggs, too, but a significant induction effect was detectable only at the end of the priming phase, i.e., 5 and 6 days after egg deposition (Figure 6B). This egg-mediated induction of camalexin persisted after egg removal for 3 and 12 h, regardless of whether plants were damaged by larval feeding (EF vs. C) or not (E vs. C; Figure 6C). Feeding damage did not affect camalexin levels, neither after 3 nor 12 h feeding.

To test whether the egg-induced camalexin levels at the end of the priming period (Figure 6B) and the persistence of enhanced levels during the feeding phase (Figure 6C) affect the larvae on previously egg-laden plants, we measured the biomass of larvae on *pad3-1* mutants (Figure 6D). Again, larvae on previously egg-laden WT plants gained less biomass than larvae on egg-free WT plants. This effect of prior egg deposition on larval biomass was still present in *pad3-1* mutants, indicating that camalexin does not play an essential role in egg-mediated priming of the plant’s defense against herbivores.

## Discussion

Our study investigated how long a plant needs to perceive insect egg deposition as “warning” of impending larval herbivory to improve (prime) its defense against the hatching larvae. Therefore, we investigated the kinetics of *A. thaliana* responses to *P. brassicae* eggs and larvae from an ecological, phytohormonal, and transcriptional perspective.

Our results show that the ecological priming effect of prior egg deposition on plant defense – here detected by the impaired larval performance – is only fully established at the end of the egg incubation period (after at least 5 days), i.e., just prior to larval hatching. Shorter egg deposition periods did not result in primed defense. This result suggested that the longer the eggs are present on the plant as “warning” of impending herbivory, the more the plant intensifies its responses. Indeed, our analysis of plant responses to eggs showed that transcript levels of several, especially SA-responsive genes significantly increased with increasing egg exposure time and reached a maximum shortly before larval hatching. Furthermore, at the end of the egg incubation time, levels of JA-Ile and camalexin significantly increased. In contrast, already early (1 day) after egg deposition, concentrations of JA and SA as well as expression of several JA-responsive genes increased and persisted at enhanced levels during the entire egg incubation time of 6 days. Our analysis of plant responses to the onset of larval feeding showed that levels of SA, JA-Ile, ABA, and camalexin were significantly higher in previously egg-laden plants than in egg-free ones. Larval performance studies on mutant plants indicate that both SA and JA-Ile might be important regulators of egg-mediated improvement of plant defense against larvae, whereas camalexin levels had no impact on the egg-mediated improvement of the plant’s response to larval feeding.

From an ecological perspective, a late and gradual increase of plant traits involved in defense against larval herbivory until the time when needed (here just prior to larval hatching) may be a cost-saving strategy. In this case, the plant invests into “getting ready for defense” only when the danger of herbivory is close. This interpretation might explain why some traits increase only late after egg deposition or gradually in the course of the egg incubation time but raises the question of why others are induced shortly after egg deposition and kept induced over (almost) the entire egg incubation time.

**Figure 7:**
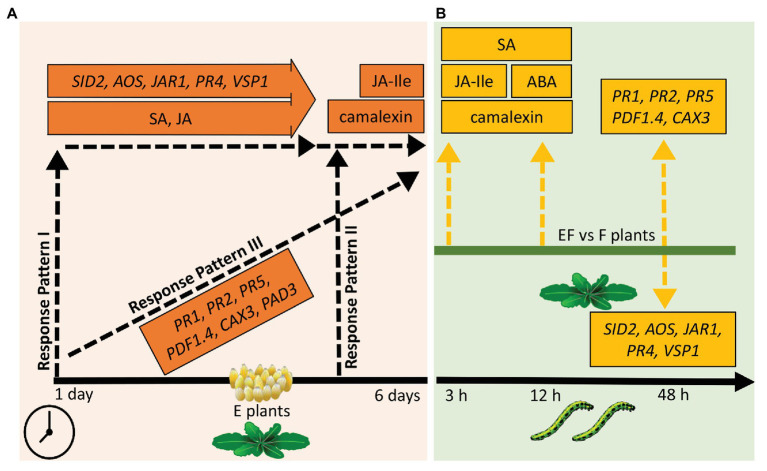
Overview of results of the phytohormone, camalexin, and gene expression measurements. **(A)** Responses of plants to eggs. Response pattern I: traits significantly increased 1 day after egg deposition, almost kept the increased level for several days but showed no further significant increase. Response pattern II: traits significantly increased only 5–6 days past egg deposition. Response pattern III: traits significantly increased shortly after egg deposition and showed further significant increase at the end of the egg incubation time. Timeline: measurements 1–6 days after egg deposition. **(B)** Responses of previously egg-laden plants to feeding. Dashed, yellow arrows pointing upwards (downwards): higher (lower) levels of measured traits in EF plants when compared to F plants; EF: plants exposed to eggs and feeding, F: plants exposed to feeding only. Timeline: measurements 3, 12, or 48 h after onset of larval feeding. Please see text for further explanation.

Our results suggest three temporal response patterns of *A. thaliana* to *P. brassicae* eggs: (i) early induced responses, which are activated shortly after egg deposition and maintained during the egg incubation time (response pattern I); such responses might contribute to defense against eggs and later hatching larvae as well as to resistance against insect-transmitted phytopathogens; (ii) responses induced late after egg deposition and protecting against impending danger of larval feeding damage and phytopathogen infection due to leaf wounding inflicted by the larvae (response pattern II); and (iii) egg-induced responses that gradually increase with the egg exposure time the closer the danger of herbivory comes (response pattern III; Figure 7A). In addition, our results suggest that egg-induced traits of response pattern I are also important for response patterns II and III against larval feeding damage and vice versa, thus providing an integrative strategy against different phases of insect infestation.

### Response Pattern I of *Arabidopsis thaliana* to *Pieris brassicae* Eggs: Early Induced and Maintained Responses

Which phytohormonal and molecular traits provide evidence for response pattern I? Levels of SA and JA were induced by egg deposition and maintained at the elevated level for the entire egg incubation time.

Salicylic acid is well-known to induce hypersensitive responses (HR) to phytopathogens (Ding and Ding, 2020, and references therein). Plants induce SA also in response to insect eggs, and this induction is associated with HR-like symptoms (e.g., Little et al., 2007; Reymond, 2013; Hilfiker et al., 2014; Bittner et al., 2017; Geuss et al., 2017; Lortzing et al., 2020), including ROS accumulation or formation of necrotic leaf tissue around the egg deposition site. These plant responses to insect eggs are known to be associated with increased egg mortality, as has been shown for *B. nigra* responding to *Pieris rapae* and *Pieris napi* eggs (Shapiro and DeVay, 1987; Fatouros et al., 2014), *P. sylvestris* responding to sawfly eggs (Bittner et al., 2017), and *S. dulcamara* responding to eggs of a moth (Geuss et al., 2017). While also *A. thaliana* Col-0 ecotype shows HR-like symptoms (chlorosis; Reymond, 2013) and ROS accumulation (Gouhier-Darimont et al., 2013), no detrimental effects of these responses to *P. brassicae* eggs are known (Griese et al., 2019). The increase of SA levels in response to eggs also mediates the plant’s protection from phytopathogens (Hilfiker et al., 2014). However, so far, no phytopathogens have been found to be associated with *P. brassicae* eggs (Paniagua Voirol et al., 2020). Even though it is well known that insects are also vectors of phytopathogens (Lynch and Lewis, 1978; Nadarasah and Stavrinides, 2011), further studies need to investigate whether the early response of *A. thaliana* to *P. brassicae* eggs may be considered a preventive response to the risk of (insect-transmitted) phytopathogen infection. While we here show that levels of free (non-derivatized) SA were already significantly enhanced 1 day after egg deposition and kept at the enhanced level for the entire egg incubation time, Bruessow et al. (2010) detected a gradual increase of total SA, which included SA-glucosides, in *A. thaliana* responding to *P. brassicae* eggs over a period of 4 days. According to our study, early (3–12 h) after the onset of larval feeding, free SA levels were also significantly higher in egg-laden plants than in egg-free ones. If larval feeding induces hydrolysis of SA-glycosides due to glucosidase activity in larval spit (Mattiacci et al., 1995), then both egg-induced free SA and SA-glycosides might contribute to the higher levels of free SA in egg-laden, feeding-damaged plants.

The egg-induced JA levels, which were kept moderately high during the entire egg incubation time, might be due to the permanent touch of the leaf by the egg cluster. Several lines of evidence suggest that “touch perception” (Weiler et al., 1993) by plants induces responses, which are mediated by JA (e.g., Tretner et al., 2008; Peiffer et al., 2009; Chehab et al., 2012). In accordance with egg-induced JA-levels, also genes involved in JA biosynthesis (*AOS*) and JA-responsive genes like *PR4* and *VSP1* (Thomma et al., 1998; Ellis and Turner, 2001) showed enhanced expression levels already at the first day after egg deposition. The ecological relevance of such an early egg-mediated induction of JA and genes involved in JA biosynthesis and signaling remains to be studied.

### Response Pattern II of *Arabidopsis thaliana* to *Pieris brassicae* Eggs: Late Induced Responses

Phytohormonal and molecular traits of response pattern II (Figure 7A) are levels of JA-Ile and camalexin. These parameters were significantly induced by the eggs only at the very end of the egg incubation time.

Enhanced levels of JA-Ile at the end of the egg deposition period may be expected to result in enhanced expression of JA-responsive genes when larvae start feeding. However, after 2 days of feeding damage, the expression of JA-responsive genes in previously egg-laden plants was even lower than in egg-free ones (Supplementary Figure S1, Figure 7B). Future studies need to analyze transcription of JA-responsive genes in the very beginning of larval feeding to further elucidate whether JA-Ile-activated expression of genes early after the onset of feeding damage is crucial for the plant’s primed defense against herbivores. Our bioassay with a *jar1-1* mutant impaired in biosynthesis of JA-Ile indicates that reduced levels of this phytohormone result in loss of the plant’s primability by insect egg deposition; larvae feeding on previously egg-laden *jar1-1* mutants gained as much biomass as larvae on egg-free *jar1-1* mutants. However, *jar1* mutants are not completely lacking JA-Ile (Suza and Staswick, 2008). Upon wounding, they still show induced expression of some JA-responsive genes; this induction may occur with a time delay when compared to the response of WT plants (Suza and Staswick, 2008). Whether delayed wounding-induced expression of JA-responsive genes has contributed to the here observed absence of the egg-mediated priming effect on the anti-herbivore defense of *jar1-1* mutant plants is unclear so far. To elucidate the relevance of JA-Ile in egg-mediated priming of defense against the larvae, follow-up studies should include larval performance studies on *coi1-1* mutant lines, which are impaired in JA-Ile perception and thus in the expression of JA-dependent genes (Devoto et al., 2005; Suza and Staswick, 2008).

Our study shows that the phytoalexin camalexin is not only inducible by phytopathogen infection (e.g., Glawischnig, 2007; Ahuja et al., 2012; War et al., 2012; Zhang et al., 2014) but also by *P. brassicae* egg deposition on *A. thaliana*. Induction of camalexin by insect egg deposition has not been demonstrated before, but other studies indicate that feeding damage by chewing herbivorous insects, e.g., by larvae of the generalist moth species *Mamestra brassicae* or *Trichoplusia ni*, also induce camalexin (Pangesti et al., 2016; Vishwanathan et al., 2020). Feeding damage by the specialist *P. brassicae* larvae for 4 days did not induce camalexin levels (Supplementary Figure S3). Furthermore, camalexin has been shown to act as a defense compound against sucking herbivores such as aphids (Kusnierczyk et al., 2008; Kettles et al., 2013). For example, in *A. thaliana* ecotype Landsberg *erecta* camalexin was induced after 48 h of feeding by *Brevicoryne brassicae* aphids, and asexual fecundity of the aphids was higher on *pad3-1* mutant plants (Kusnierczyk et al., 2008). *Arabidopsis thaliana* ecotype Col-0 responded to feeding by *Myzus persicae* with upregulation of *PAD3* within 12 h after infestation, and the fecundity of these aphids was also higher when feeding on *pad3-1* mutant plants (Kettles et al., 2013), indicating the defensive role of this compound against different aphid species. The late induction of camalexin in the end of the egg incubation period suggested that this indole alkaloid exerts adverse effects on the hatching larvae feeding upon previously egg-laden plants. However, our bioassay with a *pad3-1* mutant impaired in camalexin biosynthesis revealed that plant defense against larvae is still primable; larvae feeding on previously egg-laden *pad3-1* mutants gained significantly less biomass than larvae on egg-free *pad3-1* mutant plants. Thus, we conclude that camalexin does not play a role in egg-mediated priming of the plant’s defense against herbivores. Nevertheless, the enhanced camalexin levels established in the end of the egg incubation time and maintained in egg-laden, feeding-damaged plants (Figures 6C, 7A,B) might benefit the plant when damaged by hatching larvae. The latter inflict leaf wounds that can provide entries for bacterial disease. Camalexin is well-known as an anti-microbial agent in systemic acquired resistance (SAR) against phytopathogens (Návarová et al., 2012). Conspicuously, *P. brassicae* egg extract induces intraplant and interplant SAR in *A. thaliana*, which therefore gets more resistant against bacterial disease elicited by *Pseudomonas syringae* infection (Hilfiker et al., 2014; Orlovskis and Reymond, 2020).

### Response Pattern III of *Arabidopsis thaliana* to *Pieris brassicae* Eggs: Gradually Increasing Induced Response

Traits representing response pattern III (Figure 7A) are the SA-responsive *PR* genes, *PAD3*, *CAX3*, and *PDF1.4*; their expression gradually increased during the egg incubation time.

Accumulation of the respective PR proteins is well-known to be associated with HR induced by phytopathogens (e.g., Balint-Kurti, 2019, and references therein). Expression of these genes was found to be induced already shortly after egg deposition but reached its maximum only at the end of the egg incubation time, suggesting that this response is not only acting against the eggs but also targeting the larvae. This assumption is supported by the findings that (i) expression of *PR* genes was also higher in egg-laden, feeding-induced plants than in egg-free, feeding-damaged ones (Supplementary Figure S1 and Figure 7B) and (ii) the plant’s primability by egg deposition was lost in *pr5* mutant plants (Lortzing et al., 2019). HR-like symptoms induced by *P. brassicae* eggs in *A. thaliana* leaves include cell death and callose deposition (Little et al., 2007). Such leaf tissue probably makes it harder for neonate, tiny larvae to gain access to nutrient-rich, well digestible leaf tissue. The gradual increase of *PAD3* expression in response to egg deposition resulted in a significant increase of camalexin at the end of the egg incubation period. The functional role of the gradual increase of *CAX3* and *PDF1.4* during the egg deposition period remains unclear. These genes showed higher transcript levels in egg-laden, feeding-damaged plants than in egg-free, feeding-damaged ones, indicating that their expression levels are relevant for priming defense against feeding larvae (Supplementary Figure S1 and Figure 7B).

### Interactions of Abscisic Acid, Jasmonic Acid, and Salicylic Acid During the Insect Egg Deposition Period and After the Onset of Larval Feeding

Interestingly, levels of SA and JA-Ile were significantly higher shortly after the onset of feeding damage in previously egg-laden than in egg-free plants (Figures 4B, 7B), suggesting a fine-tuned interplay of these phytohormones in priming a plant for improved anti-herbivore defense. The fine-tuning of the hormonal interactions may depend on hormone concentration, timing of induction, and sites of induction, as outlined below.

During the egg incubation time, no antagonistic effects of the egg-induced JA and SA levels on expression of the analyzed JA- and SA-related genes were observed. These results are in agreement with a study by Mur et al. (2006) demonstrating that the outcome of the interaction of JA and SA is plastic and depends on the hormonal induction level. In our study, levels of SA increased to about 100 ng/g leaf fresh weight after egg deposition, while egg-induced JA levels were about 10-fold lower. However, 1 day after larval feeding, JA levels were much higher (Lortzing et al., 2019). Our study here showed that the expression of JA-responsive genes was upregulated 2 days after larval feeding in comparison to untreated controls (Supplementary Figure S4B) but downregulated in previously egg-laden plants when compared to feeding-damaged plants without prior egg deposition (Supplementary Figure S1B). In contrast, the feeding-induced expression of *CAX3*, the SA-responsive *PR* genes and *PDF1.4* in egg-free plants (Supplementary Figure S4) were further enhanced in feeding-damaged plants with prior egg deposition (Supplementary Figure S1A). Previous studies revealed that *A. thaliana* plants damaged by *P. brassicae* or by *Spodoptera littoralis* larvae for 48 h show suppressed induction of wounding- and JA-responsive genes, when the plants have been treated with egg extracts of *P. brassicae* prior to larval feeding (Bruessow et al., 2010; Bonnet et al., 2017). The treatment of plants with egg extracts resulted in the suppression of plant defense against larvae of the generalist *S. littoralis* but not of the specialist *P. brassicae* (Bruessow et al., 2010). A study by Schweizer et al. (2013) indicates that *P. brassicae* larvae are hardly affected by JA-mediated plant defense responses. Our current study here and previous studies show that *P. brassicae* larvae perform worse on previously egg-laden plants (Geiselhardt et al., 2013; Bonnet et al., 2017; Lortzing et al., 2019), which may be especially due to SA-mediated plant defense responses. The significance of SA for egg-mediated reinforcement of plant defense against larvae has been demonstrated already by our previous study (Lortzing et al., 2019) and is confirmed here by the bioassays with the *sid2* mutant.

Timing of induction of different phytohormones may decide how they interact. Since levels of both JA and SA were induced already 1 day after egg deposition and maintained during the entire egg incubation time, their induction by eggs was not temporarily separated. However, the moderate JA levels induced by egg deposition might have contributed to the higher levels of JA-Ile in previously egg-laden plants damaged by larvae for 3 h. A bit later after onset of feeding damage (12 h), JA-Ile levels were equally high in egg-laden and egg-free plants. This finding suggests that the plant’s response to eggs results in earlier or accelerated conjugation of JA to the active JA-Ile when damaged by feeding larvae. In addition to hormone levels and timing of induction, the sites of induction may affect hormonal interactions. A study by Betsuyaku et al. (2018) provided evidence of spatial separation of induction of JA and SA levels in response to bacterial infection. While SA accumulated at the site of infection, JA accumulated in the surroundings of the infection site. Whether such spatial separation also occurs in response to egg deposition remains to be addressed in future studies.

Our study demonstrated that in spite of the often observed antagonistic interactions of SA and JA (Erb et al., 2012; Pieterse et al., 2012; Thaler et al., 2012), both phytohormones seem to be relevant for egg-mediated improvement of plant defense against larval feeding as indicated by our bioassays with *jar1-1* and *sid2* mutant plants. Priming of both the SA- and JA-mediated signaling pathway is also known for plant defense responses to other biotic stressors than insect infestation (e.g., Martínez-Medina et al., 2017; Jia et al., 2018). For example, priming of *A. thaliana*’s defense against *P. syringae* DC3000 by chitosan simultaneously upregulates SA- and JA-marker genes and enhances levels of SA and JA (Jia et al., 2018).

A recent study comparing the transcriptomes of different plant species infested by insect eggs and larvae suggests that the interplay of several phytohormones, especially JA, SA, and ABA, are required to prime a plant for improved defense against herbivorous larvae (Lortzing et al., 2020). In the study here, not only SA and JA-Ile levels but also ABA levels were significantly higher in previously egg-laden, feeding-damaged than in egg-free, feeding-damaged plants (Figures 4B, 7B). ABA-mediated signaling may synergistically interact with JA-mediated plant responses to chewing insects (Pieterse et al., 2012; Nguyen et al., 2016; Vos et al., 2019). Our results show that an egg-mediated increase of JA-Ile levels 3 h after the onset of feeding preceded the ABA burst after 12 h feeding upon egg-laden plants (Figure 4B). Thus, ABA might be important to reinforce the plant’s defense against herbivores. However, whether ABA is also required for egg-mediated improvement of the plant’s anti-herbivore defense has not yet been experimentally proven.

## Conclusion

Our results suggest that egg-mediated priming of *A. thaliana’s* defense against herbivores is based on a fine-tuned temporal pattern of gene expression and phytohormonal signaling. Expression of the tested genes and changes of the analyzed phytohormone levels showed different kinetics. While several responses are induced already shortly after egg deposition, others are induced only late or gradually increase until the end of the egg incubation time. The egg-induced responses were shown to modify feeding-induced responses that negatively affect the herbivore. Furthermore, camalexin levels induced late after egg deposition may enhance the plant’s defensive forces against bacterial infection at the onset of larval feeding. This could be beneficial for the plant because the wounds inflicted by larval feeding may provide entries for bacterial phytopathogens.

Our study shows that the plant’s response to eggs results in amplifying some feeding-inducible defense traits against hatching larvae. Furthermore, the kinetics of changes indicates an earlier or accelerated feeding-induced change of JA-Ile levels in previously egg-laden than in egg-free plants. Such acceleration of organismic stress responses due to prior responses to danger-indicating cues may be a strategy serving improved stress management, in addition to amplification of stress responses primed by “warning” cues (Hilker et al., 2016; Hilker and Schmülling, 2019). Whether previously egg-laden plants also show earlier induction of JA-responsive genes at the onset of larval feeding than egg-free plants needs to be addressed in future studies.

## Data Availability Statement

The original contributions presented in the study are included in the article/Supplementary Material, further inquiries can be directed to the corresponding author.

## Author Contributions

GV, NB, RK, NF, MH, and VL designed the study and planned the experiments. GV and VL conducted the experiments, analyzed the data, and wrote a first draft of the manuscript. MH and VL revised the manuscript. All authors contributed to the article and approved the submitted version.

### Conflict of Interest

The authors declare that the research was conducted in the absence of any commercial or financial relationships that could be construed as a potential conflict of interest.
