# Supplementary material for: Priming by Timing: Arabidopsis thaliana Adjusts Its Priming Response to Lepidoptera Eggs to the Time of Larval Hatching
Source: Front Plant Sci. 2020 Dec 9;11:619589. doi: 10.3389/fpls.2020.619589 (PMC7755604; doi:10.3389/fpls.2020.619589)
Supplement: Supplementary file 1 [file Data_Sheet_1.pdf]

Supplementary Material

1 Supplementary Tables

Supplementary Table S1. List of analyzed genes and qRT-primers designed by PearlPrimer (Marshall 2004) and obtained from Eurofins Genomics.

		**Sequence 5´→3´**	
**Target**	**AGI**	**Forward**	**Reverse**
ACT2	AT3G18780	CTTCCCTCAGCACATTCCAG	GACCTGCCTCATCATACTCG
UBQ10	AT4G05320	GGCCTTGTATAATCCCTGATGAATAAG	AAAGAGATAACAGGAACGGAAACATAGT
GAPDH	AT1G13440	TTGGTGACAACAGGTCAAGCA	AAACTTGTCGCTCAATGCAATC
genomic control		TTTTTTGCCCCCTTCGAATC	ATCTTCCGCCACCACATTGTAC
SID2	AT1G74710	CCGTGACCTTGATCCTTTCTC	CATTAAACTCAACCTGAGGGAC
PR1	AT2G14610	ACACGTGCAATGGAGTTTGTGG	TTGGCACATCCGAGTCTCACTG
PR2	AT3G57260	CACAGCTGGACAAATCGGAG	CAAGATCTGAACTGGGAACGTC
PR5	AT1G75040	GATGTGAGCCTCGTAGATGGT	ACATTGTTCTGATCCATGACCT
CAX3	AT3G51860	CTTTCTACACTGGTCCAACAGTG	TATTTCACCACTGCCACTTTGTTA
PDF1.4	AT1G19610	ACACACTTATGCTCTTCCTTTGCC	ACCGCCATCATCTCAGTGGAAG
AOS	AT5G42650	GGTGGCGAGGTTGTTTGTGATTG	TTCCTAACGGCGACGTACCAAC
JAR1	AT2G46370	CGGTTATTTCGAGTTTCTCCCT	GATACAACCCTGCGTAATTTGTG
MYC2	AT1G32640	AACCACGTCGAAGCAGAGAGAC	TTGGTACAACCGCTCGTAACGC
PR4	AT3G04720	CGCCACCTACCATTTCTATAATCC	CTTGTGTTCTTCACCCTTAAACAC
VSP1	AT5G24780	ATCTCATACTCAAGCCAAACGG	TCCTCAACCAAATCAGCCCA
ABA1	AT5G67030	AACCTATGTGACCGATAACGA	TAAACGCCGCCTTCTTATCTG
ABI1	AT4G26080	AACTGCACTTCCATTATCCGT	AAGTATCTATCGCCAATGGATCTC
PAD3	AT3G26830	TGATCTCGGACATATTTGTAGCAG	GCACTTTCTTCATCACTCTTGG

2 Supplementary Figures

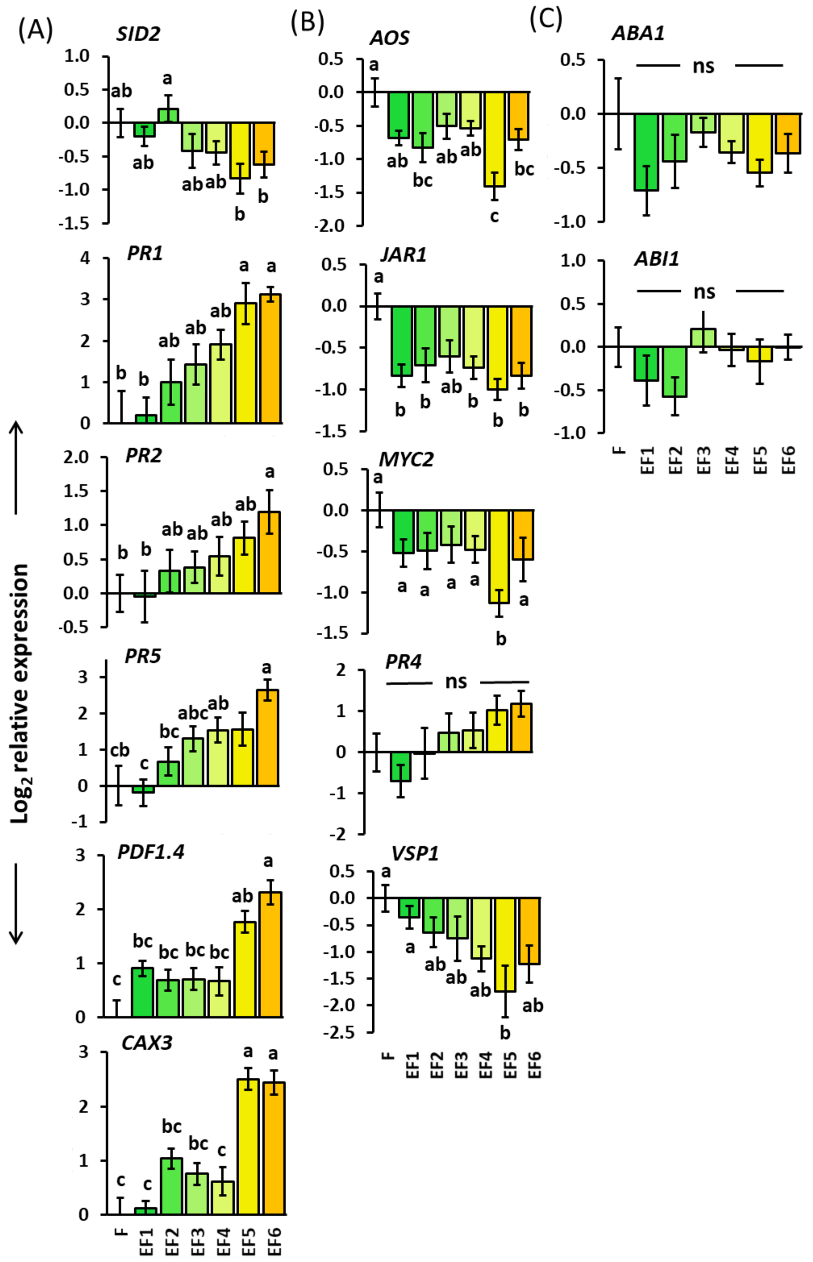

Supplementary Figure S1. Impact of increasing *Pieris brassicae* egg deposition period on transcript levels in *Arabidopsis thaliana* leaves damaged by larval feeding for 48 h. Relative expression ($\log_2$, mean ± SE) of **(A)** genes known to be involved in egg-mediated responses of *A. thaliana*, **(B)** jasmonic acid biosynthesis genes and jasmonic acid-responsive genes and **(C)** abscisic acid biosynthesis genes and abscisic acid-responsive genes in feeding-damaged, egg-free leaves (F) and in feeding-damaged leaves previously exposed for one (EF1), two (EF2), three (EF3), four (EF4), five (EF5), or six days (EF6) to *P. brassicae* eggs. Larvae fed for 48 h on treated plants. Different letters indicate significant differences between treatments ($P < 0.05$, linear mixed model and post hoc general linear hypothesis test with Tukey contrasts). Biological replicates (plants) per treatment: N = 9–10.

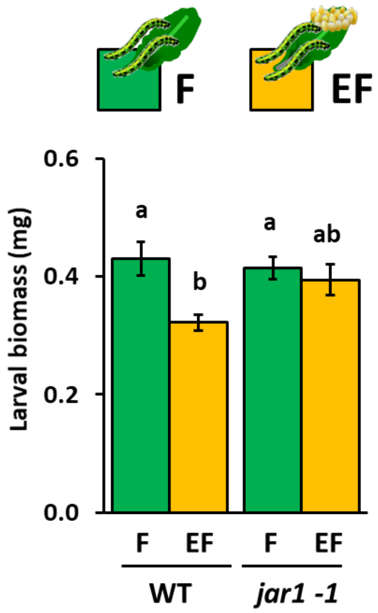

Supplementary Figure S2. Impact of *Arabidopsis thaliana* JAR1-impairment on performance of *Pieris brassicae* larvae that fed on previously egg-laden plants. Biomass in mg (mean ± SE) of *P. brassicae* after feeding for 48 h on egg-free (F, green) or egg-laden (EF, yellow) *A. thaliana* wild type (WT) or *jar1-1* mutant plants. Different letters above the bars indicate significant differences at the level of $P < 0.05$ (pairwise t-test with Benjamini Hochberg correction). Biological replicates (plants) per treatment: $N = 8$.

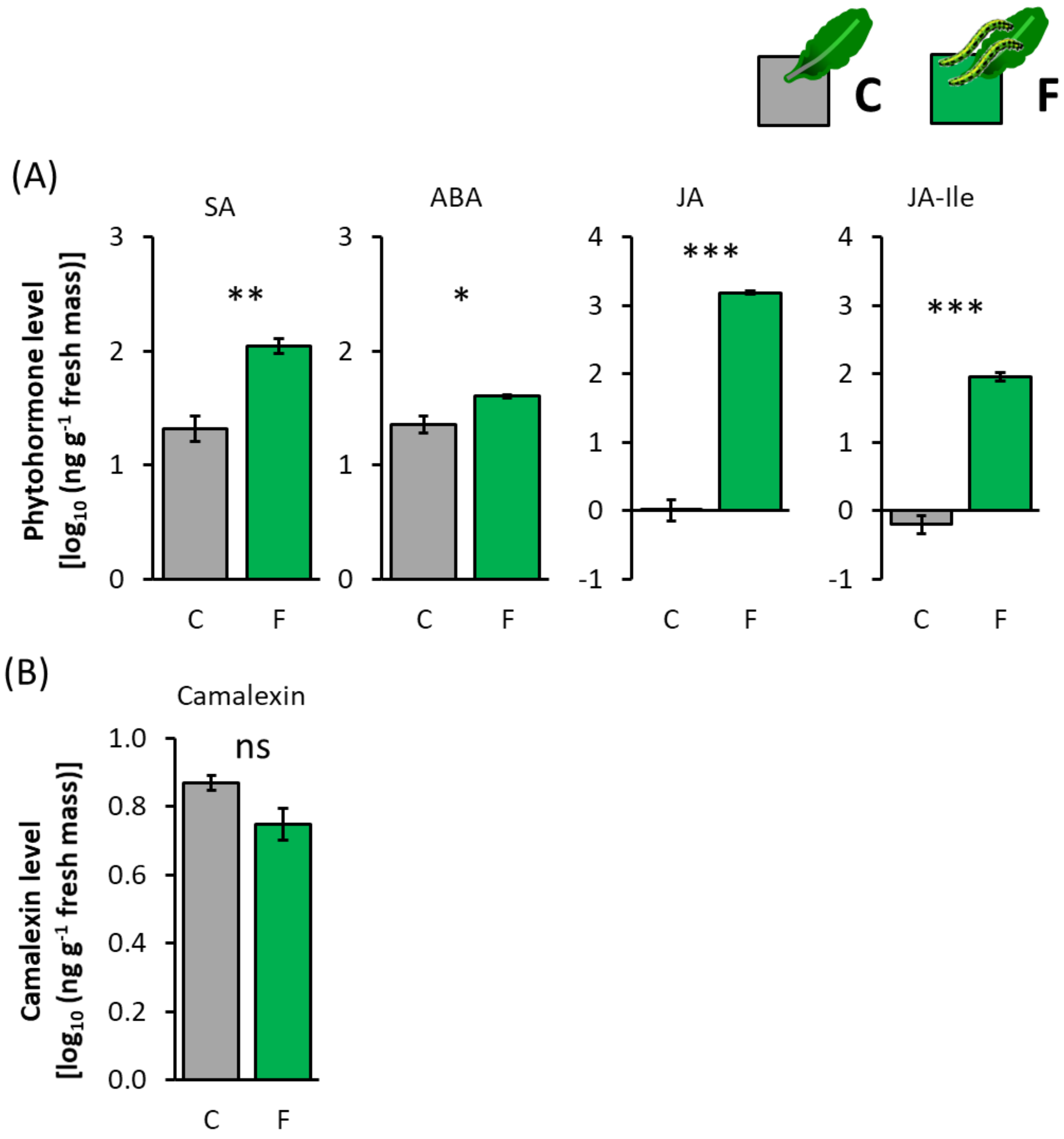

Supplementary Figure S3. Impact of *Pieris brassicae* feeding damage for 96 h on (A) phytohormone and (B) camalexin levels in egg-free *Arabidopsis thaliana* leaves (mean ± SE). Asterisks indicate significant differences between the treatments at the level ***: $P < 0.001$, **: $0.001 < P < 0.01$, * $0.01 < P < 0.05$, ns for non-significant differences ($P > 0.05$, Student's t-test). Biological replicates (plants) for each control and feeding-treatment: $N = 4$.

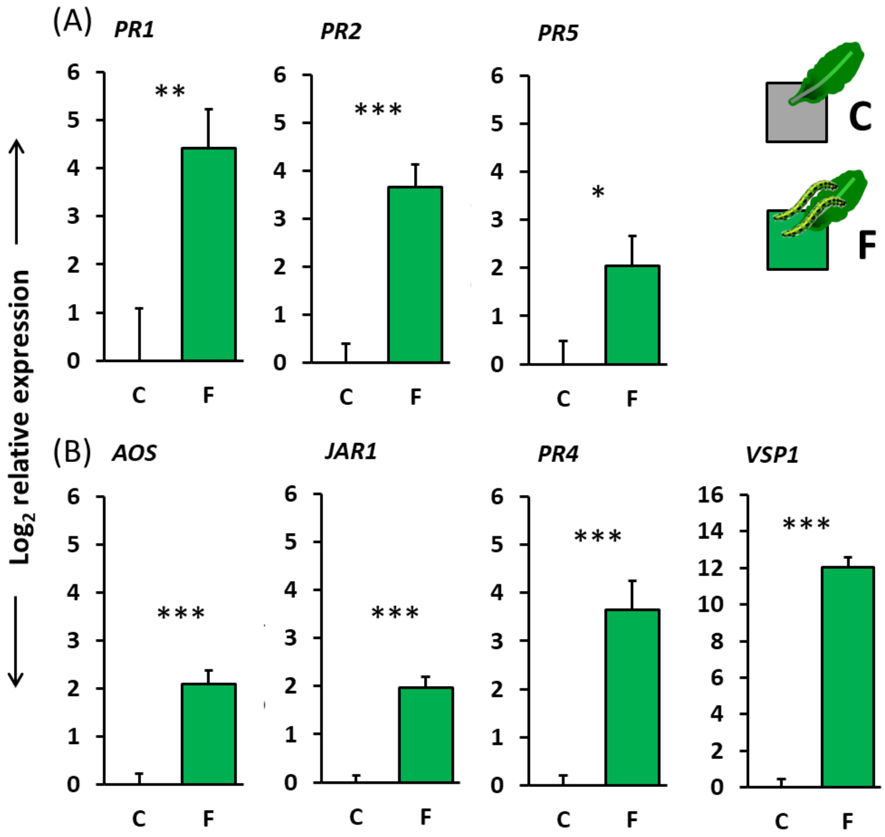

Supplementary Figure S4. Impact of *Pieris brassicae* feeding damage for 48 h on transcript levels of egg-free *Arabidopsis thaliana* leaves. Relative gene expression ($\log_2$, mean ± SE) of genes involved in salicylic acid-mediated signaling (*PR1*, *PR2* and *PR5*) and genes involved in jasmonic acid biosynthesis and signaling (*AOS, JAR1*, *PR4* and *VSP1*). Asterisks indicate significant differences between the treatments at the level ***: $P < 0.001$, **: $0.001 < P < 0.01$, * $0.01 < P < 0.05$, (Student's t-test). Biological replicates (plants) each for control and feeding-treatment: $N = 6\text{-}8$.

3 Supplementary material: Reference

Marshall, O. J. (2004). PerlPrimer: Cross-platform, graphical primer design for standard, bisulphite and real-time PCR. *Bioinformatics* 20, 2471–2472. doi: 10.1093/bioinformatics/bth254
